# Construction of nano slow-release systems for antibacterial active substances and its applications: A comprehensive review

**DOI:** 10.3389/fnut.2023.1109204

**Published:** 2023-02-01

**Authors:** Jiayong Cao, Mingkun Gao, Jian Wang, Yuan Liu, Xuan Zhang, Yi Ping, Jia Liu, Ge Chen, Donghui Xu, Xiaodong Huang, Guangyang Liu

**Affiliations:** ^1^College of Agriculture and Forestry Science and Technology, Hebei North University, Hebei Key Laboratory of Quality and Safety Analysis-Testing for Agro-Products and Food, Zhangjiakou, China; ^2^State Key Laboratory of Vegetable Biobreeding, Institute of Vegetables and Flowers, Chinese Academy of Agricultural Sciences, Key Laboratory of Vegetables Quality and Safety Control, Ministry of Agriculture and Rural Affairs of China, Beijing, China; ^3^College of Horticulture, Northeast Agricultural University, Harbin, Heilongjiang, China; ^4^Internal Trade Food Science Research Institute Co., Ltd, Beijing, China

**Keywords:** nanocarriers, encapsulation technology, assembly/release mechanism, bacteriostatic, antibacterial active substances

## Abstract

At present, nano-carrier materials with antibacterial activity are of great significance. Due to the widespread resistance of many pathogenic microorganisms, it has seriously threatened human health. The natural antimicrobial substances extracted from fruits and vegetables can significantly improve their stability combined with nano-carrier materials. The resistance of pathogenic microorganisms will be substantially reduced, greatly enhancing the effect of active antimicrobial substances. Nanotechnology has excellent research prospects in the food industry, antibacterial preservation, food additives, food packaging, and other fields. This paper introduces nano-carrier materials and preparation techniques for loading and encapsulating active antibacterial substances in detail by constructing a nano-release system for active antibacterial substances. The antibacterial effect can be achieved by protecting them from adverse external conditions and destroying the membrane of pathogenic microorganisms. The mechanism of the slow release of the bacteriostatic active substance is also described. The mechanism of carrier loading and release is mainly through non-covalent forces between the bacteriostatic active substance and the carrier material, such as hydrogen bonding, π-π stacking, van der Waals forces, electrostatic interactions, etc., as well as the loading and adsorption of the bacteriostatic active substance by the chemical assembly. Finally, its wide application in food and medicine is introduced. It is hoped to provide a theoretical basis and technical support for the efficient utilization and product development of bacteriostatic active substances.

## 1. Introduction

The rapid emergence of antibiotic-resistant pathogens seriously threatens human health (1). Therefore, developing natural, efficient, low-toxic antibacterial products has become a research hotspot. Natural antibacterial active substances are essential resources to maintain human health. There are three main natural antibacterial active substances: natural plant antibacterial agents (carvacrol, eucalyptol, thymol, etc.) (2, 3). Natural animal antimicrobials (high molecular carbohydrates, natural peptides, and amino acids) (4, 5) and natural mineral antimicrobials (clay, etc.) (6). Unlike chemical antimicrobials, which are widely used, natural products are the primary source of bioactive substances. Due to their structural characteristics and chemical heterogeneity, they can play a crucial role in disease treatment and drug innovation (7, 8), and have superior performance in the food industry and packaging.

Antibacterial active substances (such as anthocyanins, sulforaphane, etc.) extracted from vegetables generally have good antibacterial, fresh-keeping, anti-oxidation, anti-cancer, anti-inflammatory, and other active functions, and are widely used in food and pharmaceutical industries (9, 10). Among them, since the active substances extracted from vegetables have the characteristics of environmental protection, no pollution, no residue, low toxicity, and side effects, antibacterial and antioxidants have become the research focus of researchers in recent years. Therefore, a large number of active substances have been developed and applied in the fields of food preservation, natural preservatives (11), and anti-inflammatory and bacteriostatic (12). Nevertheless, the existing active substances and active substance products have certain disadvantages, such as unstable product properties, easy decomposition, and poor water solubility, resulting in the practical application effect is not outstanding.

**Figure 1:**
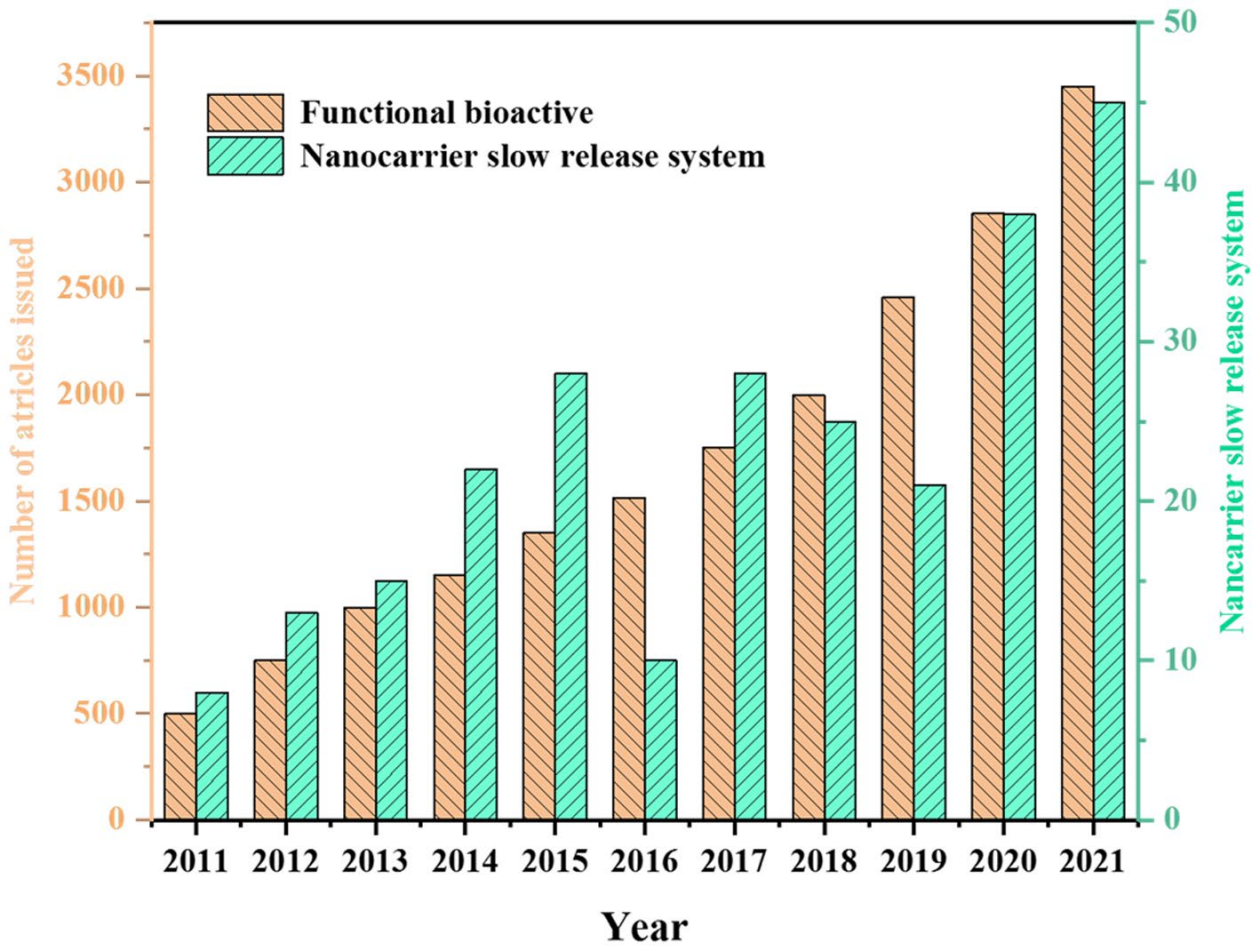
Number of publications for the term Functional bioactive and Nanocarrier slow-release system in the last decades (data collected from the Web of Science).

Meanwhile, with growing interest in nanotechnology, researchers are eager to use nanotechnology to design drug-release delivery systems that limit, protect, and specifically release functional bioactive substances, thereby synergistically improving the antibacterial effect of active substances (Figure 1). In particular, nanotechnology-based slow-release systems for biological ingredients nanocarriers have nanoscale dimensions, generally between 50 and 300 nm compared with traditional pharmaceutical formulations. They can also control the kinetics of the reaction. This will facilitate the application of nano-delivery systems in various fields such as medicine, agriculture, and food (13). In this paper, firstly, the carrier materials and encapsulation techniques of different nano-release systems were briefly introduced. Secondly, the release and action mechanism of different nanocarriers and their applications in bacteriostasis and antioxidant preservation fields were reviewed. Finally, the problems and future challenges in the delivery of nanocarriers were discussed. This review aims to highlight the applications of nano systems in functional bioactive substances and their potential opportunities and challenges.

## 2. Construction of nano slow-release system

**Figure 2:**
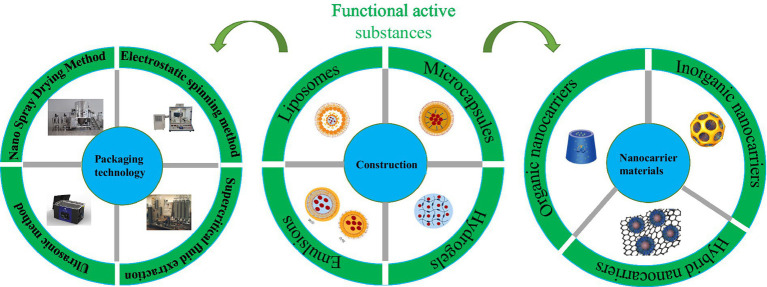
Construction of nano slow-release system.

Stability is critical to ensuring bioactive compounds, but most bioactive compounds exhibit poor stability. In this case, bioactive compounds can be loaded by selecting a suitable nanocarrier slow-release system that allows them to be transported to specific receptors to exert their efficacy (14). In addition, nanoencapsulation techniques can be used as a means to control stability and solubility to ensure the perfect release of bioactive components. Figure 2 shows the construction of a nano slow-release system.

### 2.1. Nano-encapsulation materials

Nano-encapsulated materials for the antibacterial active substances can be divided into three main categories: inorganic nanocarrier materials, organic nanocarrier materials, and hybridized nanomaterials. Inorganic nanomaterials mostly refer to metallic materials, such as carbon nanomaterials, gold and silver nanoparticles, quantum dots, and other materials. Organic nanomaterials refer to various proteins and polysaccharides, such as wheat alcohol-soluble protein, corn alcohol-soluble protein, modified starch, pectin, and other substances. In contrast, a hybrid nanomaterial is a mixture of two nano-or molecular-level components. In nature, it is usually a mixture of an inorganic and an organic substance. So, they differ from the usual sense of a mixture in that the usual mixture is of macroscopic magnitude (microns or millimeters). Mixing at the microscopic level can result in a homogeneous mixture of substances that will exhibit properties intermediate to the two components or even some new properties. Therefore, the choice of each type of nanocarrier is limited by its bioavailability, ease of application, biodistribution, food matrix compatibility, biodegradability, and biocompatibility (15). Thus, the different antibacterial active substances can be encapsulated by choosing suitable nanomaterials according to the above properties.

#### 2.1.1. Inorganic nanocarriers

Inorganic nanomaterials are a class of nanoparticles with various morphologies and particle sizes ranging from 1 to 100 nm that can be synthesized by physical or chemical methods. Inorganic nanomaterials are not only easy for surface modification but also can be bound to drug molecules in different ways. For example, electrostatic interactions, hydrophobic interactions, covalent bonding of enzyme-sensitive groups, etc. This leads to responsive release, making them ideal for drug delivery. Compared with other nanomaterials, inorganic nanomaterials have the advantages of easy preparation, high drug loading rate, controllable shape and size, good biocompatibility, and easy surface modification for drug delivery (12, 16). Therefore, it is possible to achieve an enhanced antibacterial effect.

##### 2.1.1.1. Carbon nanotubes

The unique biological and physicochemical properties of carbon nanotubes (CNT) make them an ideal nanocarrier material for the biologically antibacterial active substance. It is a tubular hollow structure containing sheets of graphene rolled together at discrete and specific angles. Depending on the number of graphene sheets rolled together, carbon nanotubes are classified as single-walled and multi-walled carbon nanotubes. These tubes can have a cross-sectional diameter of 0.4–100 nm, while the length of the tube extends thousands of times the diameter. In nano-delivery systems, such carbon nanotubes have a high aspect ratio, ultra-light specific surface area, nano-needle-like structure, and a wide range of application prospects (17). The structural stability, flexibility and surface modification of functionalized nanotubes make them good carriers of active substances. Under this concept, functionalized carbon nanotubes are widely used to encapsulate or connect antibacterial active substances. The carbon nanotubes also have superior antibacterial properties (18). To enhance the antibacterial activity of multi-walled carbon nanotube (MWCNT), modification of MWCNT is necessary. Chen Yuan et al. (19) applied a nanohybrid comprising silver nanoparticles within dendritic poly(amidoamine) dendrimer-modified MWCNT as an antimicrobial solution against Gram-negative and Gram-positive bacteria.

Hence Ghahremani was inspired by mussels to design a novel nanocarrier consisting of polydopamine (PDA), chitosan (CH) and zinc cations modified on the surface of multi-walled carbon nanotubes (MWCNT) (20). PDA, CH and Zn^2+^ were found to be successfully modified on the oxidized multi-walled carbon nanotube (OMWCNT) surface employing characterization. The material was incorporated into an epoxy coating to form a nanocomposite that exhibited superior barrier capabilities and provided stable corrosion inhibition for nearly 9 weeks.

##### 2.1.1.2. Au nanoparticles

Au nanoparticle (AuNP) carriers construct an effective delivery medium that can be applied in different fields, and gold nanoparticles have different anisotropic properties such as nanostars, nanorods, nanocages, nanoshells, and nano prisms (21–23). Among the many properties of gold nanocarriers, their optical properties are the primary factor that attracts them to biomedical and food applications. It enables the attachment of different biomolecules to gold nanoparticles. Such as enzymes, carbohydrates, fluorophores, peptides, proteins and genes. This enables efficient intracellular transport of molecules to overcome relevant barriers (24).

Au nanoparticles revealed a noteworthy antimicrobial effect against Gram-positive and Gram-negative bacterial strains. Usually, the negatively charged surface of the bacterial cell wall is attracted to the positively charged nanoparticles due to the electrostatic force of the interaction (25). In contrast, gold nanoparticles create strong bonds with the bacterial cell membrane, leading to the rupture of the cell wall and cell membrane, which leads to the disruption of biological processes. The bonds formed between metal ions and biomolecules are reported to be indeterminate and gold nanoparticles exhibit broad-spectrum activity (26, 27).

The advantages of synthesizing gold nanoparticles from active substances extracted from vegetables are not limited to the reduction of environmental toxicity, the production of nanoparticles in large quantities, the simplicity of reducing metal salts, the rapidity, the economic efficiency, the safety of clinical studies, and the nanoparticles are other advantages associated with this synthetic method. Aderonke used active substances extracted from vegetables in combination with gold nanoparticles to prepare a nanoparticle with good antibacterial effect. The percentage inhibition zones of the synthesized gold nanoparticles against the tested fungi and bacteria ranged from 30 to 66% and 40 to 54%, respectively (27).

##### 2.1.1.3. Quantum dots

Quantum dots (QD) include atoms in the II-VI (Se, Zn, Te, Cd) or III-V (in, As, P) elemental groups of the periodic table. They are colloidal nanocrystals and energy donors. The size of the quantum dots changes the luminescence between the UV–NIR region, i.e., smaller quantum dots (2 nm) fluoresce blue and larger ones (5 nm) fluoresce red (28). This optical property with long-time light emission and less photobleaching makes it superior to other organic dyes, which allows it to be used for cell imaging. Normally, the toxic cadmium in Cd Se quantum dots is encapsulated within a ZnS shell to protect it from toxicity. This enhances the accumulation of nanoparticles at the desired vascular sites. These quantum dots are also considered to be efficient delivery and reporting systems.

#### 2.1.2. Organic nanocarriers

##### 2.1.2.1. Polysaccharides

Chitosan is a polycationic polymer derivative of chitin, prepared by alkaline deacetylation of chitin. Besides cellulose, chitosan is the most abundant biopolymer existing in nature, and its structure consists of-d-glucosamine deacetylation unit and n -acetyl- d -glucosamine acetylation unit linked by β- (1, 4) glycosidic bond. Chitosan and its nanoparticles have good bioactivity and loading capacity and have been gaining more and more attention in pharmaceutical and functional food applications (29), where it has been found that the nanoparticles not only have good loading capacity for antibacterial active substance but also have outstanding slow-release properties (30).

##### 2.1.2.2. Starches

Starch has been widely used in food, textile and pharmaceutical fields under its wide source, low cost and high biocompatibility (31, 32). However, natural starch has a large relative molecular weight, poor enzymatic resistance, and does not contain hydrophobic groups, so direct preparation of nanocarriers from natural starch for functional factor encapsulation has a limited application. The starch structure can be easily modified and its molecular fine structure can be targeted and regulated according to the properties of functional factors to prepare starch nanocarriers to achieve effective encapsulation. The starch nanocarriers can be prepared for the purpose of effective embedding. Starch nanocarriers are generally in the form of starch nanoparticles, starch nanocrystals (33), starch nanofibers (34), and nanostructured starch. The starch nanocarrier forms generally include starch nanoparticles, starch nanocrystals, starch nanofibers and nanostructured starch.

##### 2.1.2.3. Protein

Protein is a biological macromolecule with multiple moieties and is characterized by a variety of chemical and physical spatial structures that can interact with a variety of food functional factors. Therefore, proteins are excellent carriers for the delivery of food active ingredients. Therefore, proteins are excellent carriers for the delivery of food active ingredients (such as curcumin), and they are also hot materials for the research of food colloidal delivery systems in recent years. The most widely used protein nano-delivery systems are animal proteins, plant proteins, and protein peptides.

#### 2.1.3. Hybrid nanocarriers

A hybridized nanocarrier is a nanocarrier that combines two or more organic nanocarriers and inorganic nanocarriers together or separately. It includes organic–inorganic, inorganic–inorganic, and multi-component. For example, lipid-polymer hybridization, ceramic-polymer hybridization, etc. combine two nanoparticles and the nanocarrier will have the dual properties of both nanoparticles, thus improving its performance many times.

##### 2.1.3.1. Metal–organic framework material

**Figure 3:**
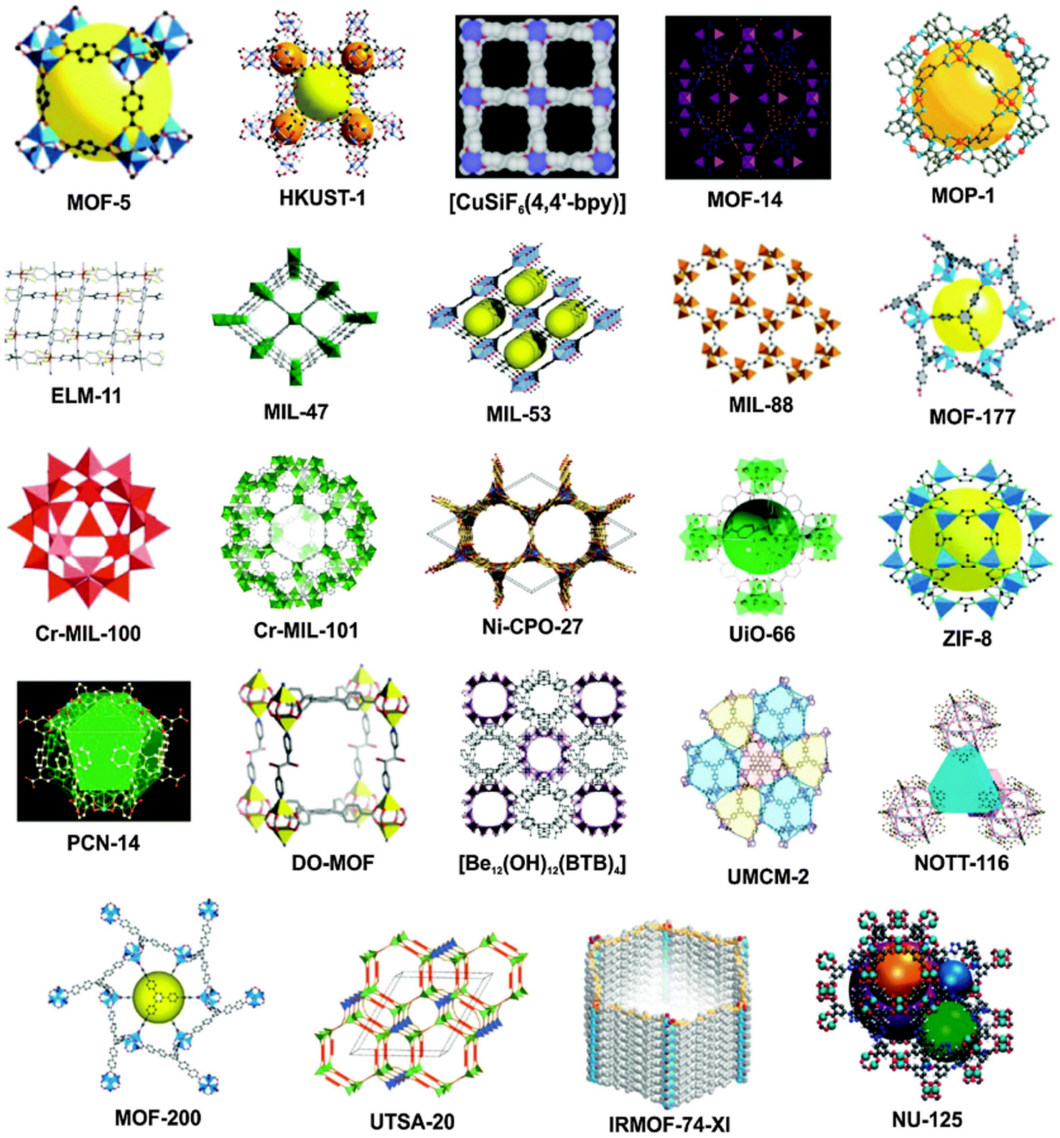
Typical examples of different types of porous MOFs structures (35).

Metal–organic framework materials (MOFs) are crystalline materials with a three-dimensional mesh structure formed by inorganic substituents (metal clusters, metal ions, central chains) and organic ligands, through coordination bonds. MOFs are an important component of hybrid nanomaterials. The most commonly used MOFs material sofistitute Lavoisier frameworks (MILs series), isoreticular metal–organic frameworks (IRMOFs series), zeolite imidazole frameworks (ZIFs series), etc. As Figure 3 shows the typical material diagram of different kinds of MOFs. Compared with traditional porous materials such as activated carbon (AC), silica gel and molecular sieve, MOF materials have the advantages of high porosity, high adsorption capacity, large specific surface area, and high modifiability. In recent years, MOF materials have been used in drug encapsulation (36), biocatalysis (37), antibacterial preservation (38), etc. MOF materials can meet the requirements of high biocompatibility, degree of dispersion and targeting required for food biomedicine through surface modification, synthesis methods, ligand changes, etc.

For example, Zeinab combined curcumin with MOF materials to construct cyclodextrin-metal organic backbone nanomaterials (CD-MOFs). The stability of curcumin was improved by three orders of magnitude and exhibited superior active functions (39). Xiao ligated HKUST-1 with folic acid to make a hydrogel wound excipient, which played a better bactericidal effect and promoted wound healing. In summary, MOF materials are promising and valuable for research in food biomedical applications, and their versatile structures and multiple applications provide new ideas for solving some problems (40).

##### 2.1.3.2. Hydrogel

Hydrogels are three-dimensional solid networks made of physically or chemically cross-linked hydrophilic polymeric structures that can entangle large amounts of water or other biological fluids within their networks (41). The formation of hydrogel nanoparticles involves the self-assembly between different charged polymers through electrostatic interactions. In their production process, the liquid phase is gelled by temperature regulation, cross-linking agents or acidification or the addition of multivalent ions. They can encapsulate hydrophilic and lipophilic bioactive, prevent degradation and target release efficiently.

Nano-hydrogels have an extremely important role in the field of drug slow release, specifically in reducing the concentration fluctuation of drug solution and releasing the loaded drug smoothly. Adila prepared a carvacrol-containing hydrogel (GG-Carv), the combination of which promotes bioavailability and delivery while maximizing the antibacterial properties of carvacrol (42). Table 1 shows the Comparison and application of different nanomaterials.

**Table 1:** Comparison and application of different nanomaterials.

Nanocarriers		Size (nm)	Encapsulated methods	Zeta potential (mV)	Applications	References
Inorganic nanocarriers	FA-GO	20–35	Self-assembly	Not reported	Cervical cancer cells	(43)
	MWCNTs-GQDs	8 ± 2	Ambient stirring	Not reported	Colon cancer cells	(44)
	MWCNTs/Au@Ag	28	Self-assembly	−10.5–22	Cancer cells	(45)
	SiO_2_/DOX	22.15	Self-assembly	Not reported	Cells	(46)
	Fe3O4 /SPMGO	9.3 ± 2.7	One-pot method	20.5–32	Cancer cells	(47)
	MSNs	199.3 ± 5.4	covalent cross-linking	−8.7 ± 0.5	Colon cancer	(48)
	F-SiO_2_@MPDA-AuNPs	20	Self-assembly	−43.7	Cancer cells	(49)
Organic nanocarriers	ZnBPMP-chiNC	176 ± 19	Photo-crosslinking	Not reported	Inflammatory Bacteria	(50)
	BLN -Wbe	140	Self-assembly	−28–50	Skin diseases	(51)
	ApoRBF	33.9	Self-assembly	Not reported	Ferritin	(52)
	OSA-modified starch	100–200	Self-assembly	Not reported	β-Carotene	(53)
	CS-GA	183–295	One-pot method	20.5–50.5	Saffron	(54)
	OCT/AVO/NLC	100–160	Photo-crosslinking	34–57	Medicinal Cosmetics	(55)
	Whey protein	96.2	Self-assembly	Not reported	β-Carotene	(56)
Hybrid nanocarriers	Pectin-chitosan	32.5	Heat-induction	Not reported	Curcumin	(57)
	Casein- zein- pectin	125.2	Chemical crosslinking	−10.5–30.5	Curcumin	(58)
	Zein-casein	32.5	pH-induction	Not reported	Adriamycin	(59)
	Cellulose	50.1	Chemical crosslinking	28.7–35.2	Drug delivery	(60)
	Chitosan	40.8	Heat-induction	Not reported	Drug delivery	(61)

### 2.2. Nano-packaging technology

#### 2.2.1. Top-down methods

##### 2.2.1.1. Emulsification method

Emulsification is the process of uniformly dispersing one liquid in very small droplets in another liquid that is immiscible (or partially immiscible) with each other. The organic polymer solution is homogenized with the aqueous phase and the solvent is then evaporated, leading to the precipitation of polymer molecules and the formation of nanospheres. This process of emulsification leads to nano emulsions loaded with active substances.

The polymer solution containing biological compounds is emulsified in the aqueous phase and then the solvent that previously dissolved the polymer is vaporized to produce nanospheres of precipitated polymer. The advantage of this method is that it is suitable for both hydrophilic and hydrophobic compounds with good solubilization ability, while the disadvantage is that this process requires a large amount of surfactant (62, 63). It provides a higher loading capacity for lipophilic compounds. Hydrophilic antibacterial active substances are poorly trapped and more difficult to scale up (64).

##### 2.2.1.2. Extrusion gelation method

Squeeze gelation is a process in which a biopolymer solution is passed through a nozzle into a gelling environment. On a small scale, the biopolymer solution is loaded into a syringe and passed through a needle into the gel state to form a gel. The technique is also suitable for industrial scale and is a gentle and convenient method for the encapsulation of hydrophilic and hydrophobic compounds (65).

Zeng introduced catechins (CC) and proanthocyanidins (PC) into rice starch gels by extrusion gelation to explore the effects of polyphenol molecules on the structure of starch gel systems in hot extrusion 3D printing (66). Owing to the strong intermolecular interactions between polyphenol molecules and starch chains, the starch gel network structure was partially disrupted, leading to a decrease in viscosity and thus improving the extrudability of the starch gel.

##### 2.2.1.3. Nano spray drying method

Spray drying is one of the most popular embedding techniques for a variety of health food products. It can be used directly for hydrophilic ingredients or indirectly for oleophilic ingredients. This technique is beneficial for many applications because it converts the liquid feed into a dry powder, which usually has higher storage stability and is also easier to transport and store (67). In brief, the process of spray drying is that the fluid material is first pumped through the atomizer, which breaks it down into a stream of fine droplets, which are then rapidly dried by the heated gas flowing through the chamber, where the droplets dry and fall to the bottom of the chamber in a few seconds, and the resulting powder is drawn into the cyclone, which is connected to an outlet fan, and the dried air is passed through a filter to separate the very fine powder, which is then removed from the entire embedding process is completed with the discharge from the spray dryer. The main challenge of this technique is the viscosity of the biopolymer suspension, which may clog the atomizer and reduce the encapsulation yield (68). Application of modified chitosan (e.g., glycol chitosan) has been proposed to obtain better nanoparticles by this method (69).

#### 2.2.2. Bottom-up method

##### 2.2.2.1. Supercritical fluid technology

Supercritical fluid technology is a new type of nanocarrier preparation technology. A supercritical fluid is a special state of fluid formed when the material is above its critical temperature and critical pressure (70). Carbon dioxide, nitrogen, methane, water, and other common gases and liquids can be used as supercritical fluids, and in the industrial application of supercritical fluid technology, the most used is supercritical carbon dioxide (SC-CO_2_). SC-CO_2_ is a new solvent with broad industrial application prospects (71). In addition, SC-CO_2_ can be used not only as a solvent, antisolvent, and solute but also in applications in drug delivery, such as the formulation of polymeric nanocarriers in combination with different drug molecules. With its tunability above the critical pressure and temperature, it provides control over particle size, particle morphology, and drug loading (72).

Curcumin is an excellent antibacterial active substance and its antibacterial effect can be improved by combining it with different nanoparticles. Xie (73) prepared curcumin nanoparticles by supercritical CO_2_, and this nano preparation This nanoparticle preparation showed higher solubility and enhanced antibacterial, antioxidant, and anticancer effects with a minimum inhibitory concentration (MIC) 50% lower than that of free curcumin solution.

##### 2.2.2.2. Nanoprecipitation

Nanoprecipitation (FNP) relies on the spontaneous emulsification of the organic internal phase of the polymer or bioactive compound and organic solvent, accommodating the external phase that then enters the aqueous phase (74). The nanoprecipitation method prepares nanoparticles by controlled mixing of solute solution and non-solvent with rapid reaction and is suitable for the preparation of a wide range of nanoparticles. Ahmed (75) used FNP to encapsulate β-carotene in NPs *via* a food-grade protein (sodium caseinate). FNP showed significant advantages in controlling NP size and increasing β-carotene loading and stability. The internal structure of NPs can be tuned by the flow rate. The loose structure is accompanied by a high release of β-carotene, while the compact structure is accompanied by a slight release.

##### 2.2.2.3. Sequential deposition

Sequential Deposition is the sequential processing of the donor and acceptor layers, thus allowing, on the one hand, the two active components to be individually regulated and optimized; on the other hand, weakening the interactions between the two components and reducing the complexity of the film formation kinetics, thus providing new opportunities for reducing production requirements. Widely used in the development of multilayer nanocarriers, with low cost, easy adoption, simple assembly techniques and better protection of lipophilic compounds (76).

Multilayer coconut oil–water emulsions containing curcumin were prepared and stabilized using a layer-by-layer technique assembled using multilayer gelatin, gum Arabic and tannic acid. Thus, the ability to control interfacial film properties by combining electrostatic, hydrophobic and hydrogen-bonding interactions may be able to control the physical stability, payload retention and accessibility of multilayer emulsions.

## 3. Loading and release mechanism of nano-delivery

The nano slow-release system can be used as a carrier to load antibacterial active substances. After reaching the corresponding target environment, there can be different release rates depending on the type or ratio of the carrier material. By adjusting the carrier material’s type or ratio, the bioactive substances’ release rate can be controlled, thus producing a nano slow-release system with targeting and slow-release characteristics.

There are two main purposes for carrier loading of the antibacterial active substance: one is to improve the situation of poor water solubility and low bioavailability of functional and active substances, and the other is to achieve a slow-release effect and improve the therapeutic effect of functional and active substances. The slow-release refers to the slow release of the drug at a predetermined rate by maintaining a constant drug concentration level for a specific time and system, reducing fluctuations in the drug concentration in the body to reduce the side effects of the drug. This slow-release system ensures that the antibacterial active substance is maintained in the effective concentration range for extended periods. The loading and release mechanism of the carrier is mainly through non-covalent forces between the antibacterial active substance and the carrier material, such as hydrogen bonding, π-π stacking, van der Waals forces, electrostatic interactions, etc., as well as the loading and adsorption of the antibacterial active substance by the chemical assembly.

### 3.1. Covalent bonding

**Figure 4:**
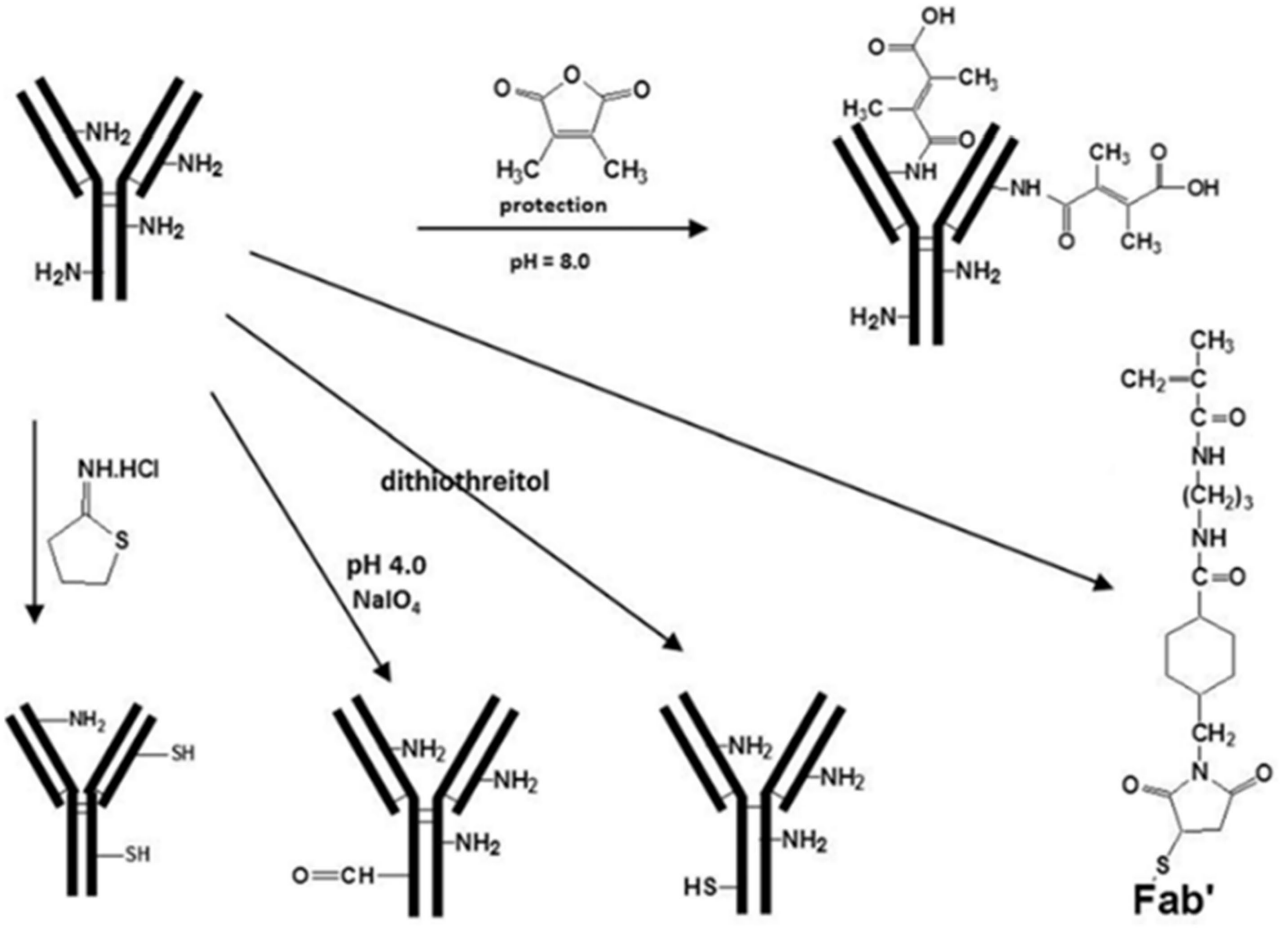
Structural modifications for covalent binding reactions of polymer precursors (77).

Nano slow-release systems can incorporate high concentrations of therapeutic agents through the presence of appropriate functional groups on their surfaces. The drug is covalently coupled to the nanocarrier due to the presence of a large number of functional groups on the surface of the nanocarrier. The release of the drug after binding occurs through enzymatic cleavage or chemical breakage of readily hydrolyzable bonds. The nanocarrier-drug coupling slowly diffuses into the cell membrane, resulting in a specific and controlled release of the drug to the target site. This covalent coupling allows stable nanocarrier systems to be used for targeted drug delivery. For instance, cross-linking and reduction of ab-binding activity can be minimized by reacting with dimethyl maleic anhydride (DMMA) and preventing them from participating in aminolysis coupling reactions. At the end of the conjugation reaction, the protecting group can be removed from the final conjugate by simply lightly acidifying the solution. By using a polymer carrier with only one reactive group at the end of the polymer chain, branching during the conjugation reaction can be avoided (Figure 4).

### 3.2. Encapsulation

**Figure 5:**
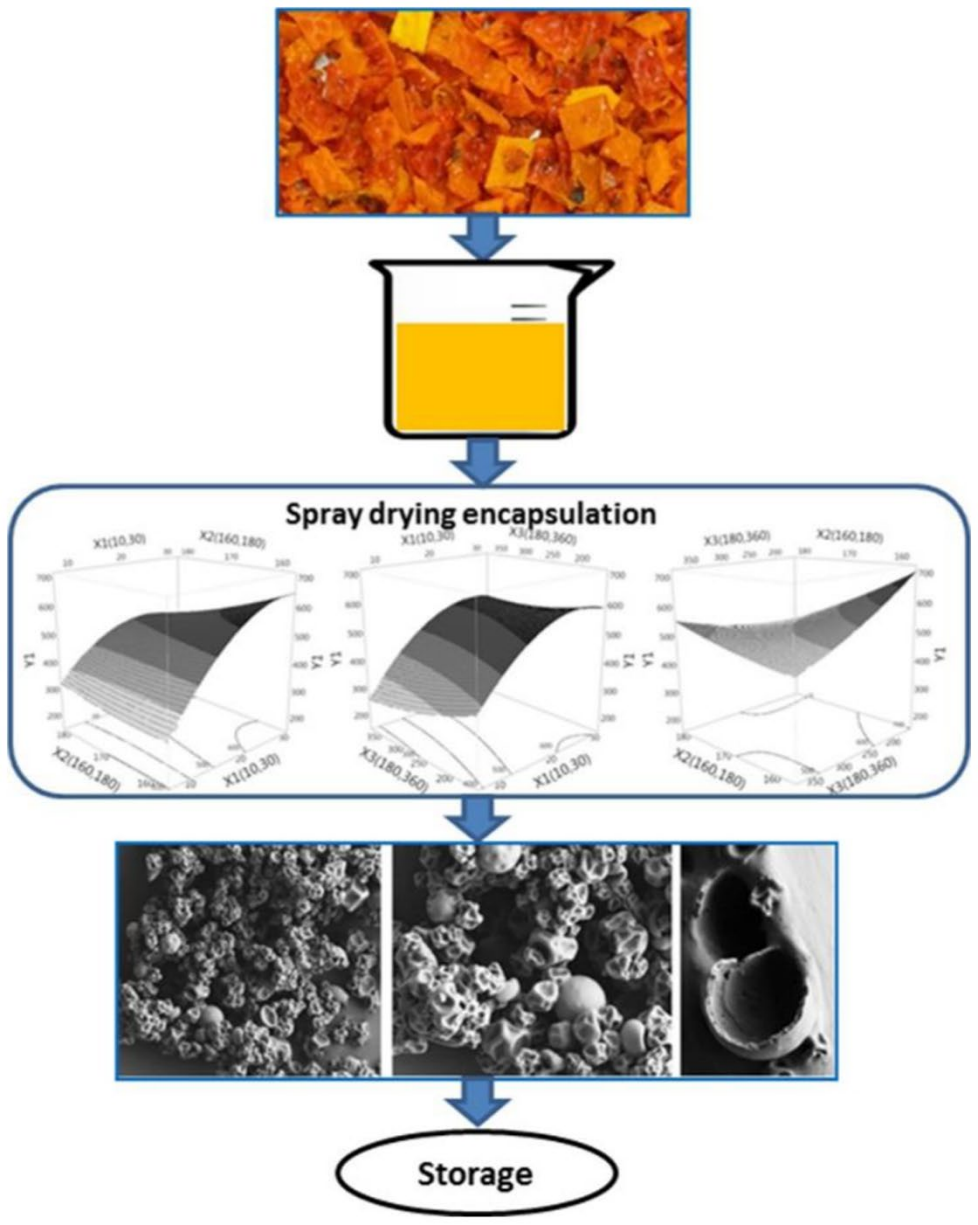
Encapsulation process of carotenoids (80).

Encapsulation is a strategy for loading therapeutic drugs into nanocarrier systems. The hollow space within the nanocarrier is capable of adequately encapsulating the drug molecules. Nanocarriers such as polymeric nanocarriers, β-cyclodextrins, nanocapsules, dendrimers, etc. can effectively encapsulate drugs within their hollow cavities (78, 79). The hydrophobic nature of the inner cavity allows the incorporation of more hydrophobic drugs in the nanocarrier through hydrophobic interactions or hydrogen bonding. This encapsulation can also occur through physical interactions. The carotenoid-rich oil recovered by Chuyen from the rind of gac fruit, a waste product of gac fruit processing, has been encapsulated using a spray dryer with a mixture of whey protein concentrate and gum Arabic. As shown in Figure 5. The encapsulation of the carotenoids by spray drying technology significantly improved the stability of the carotenoids and extended the storage period, while also providing them with a slow and controlled release effect (81). In the case of liposomes, encapsulation occurs through active and passive drug loading. The release of the drug takes place through pH-tendency neutralization or hydrolysis, thiolysis and thermal decomposition mechanisms (81).

### 3.3. Electrostatic interactions

**Figure 6:**
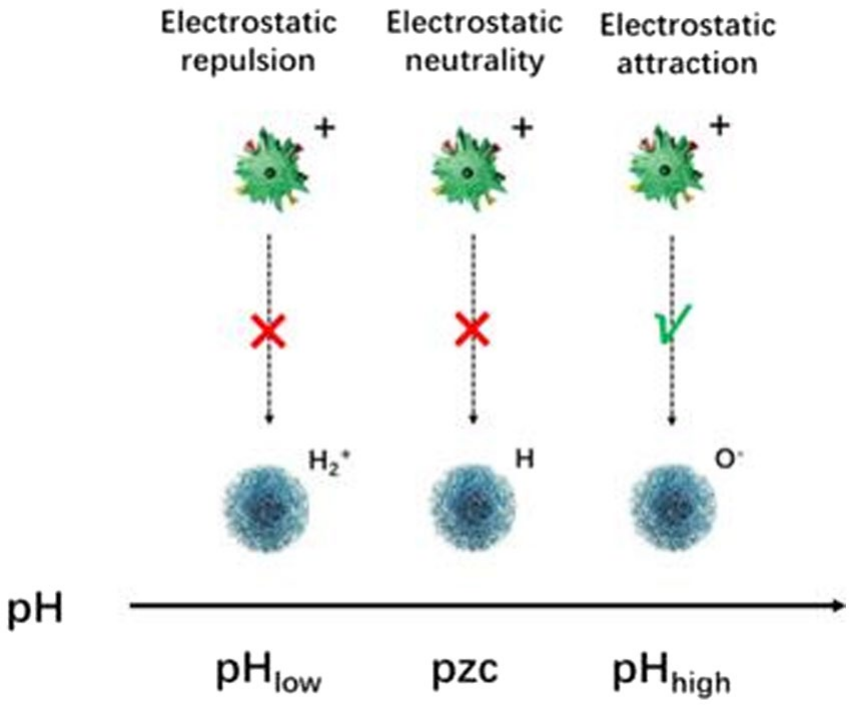
Schematic diagram of electrostatic interaction under different pH conditions.

Electrostatic interactions are the most direct loading strategy, while the adsorption of active substance molecules depends mainly on the surface potential of the nanocarrier material and the type of active substance structure. In general, the adsorption behavior of common nanocarriers is mainly influenced by the pH value. When the solution pH is low, i.e., the solution is acidic, a large number of hydrated hydrogen ions in the solution will compete with the metal ions on the surface of the material for available adsorption sites, and the positively charged surface of the nanocarrier material at this time does not have the electrostatic gravitational force and cannot load the functional active substance well into the pore structure of the nanocarrier material. On the contrary, when the solution pH is high, electrostatic attraction exists between the active substance and the metal ions in the pores of the carrier material (Figure 6).

Nanocarriers with functional groups such as carboxyl and amine groups increase the solubility of hydrophobic drugs, and these high-density functional groups enable electrostatic interactions between antibacterial active substance and nanocarrier materials, which in turn encapsulate them in the nanomaterials to form a nano-delivery system. Certain NSAIDs, such as indomethacin, ciprofloxacin, diflunisal, and ibuprofen, are effectively integrated within the nanocarriers through electrostatic interactions, which greatly improves the therapeutic efficacy of these drugs (82).

**Figure 7:**
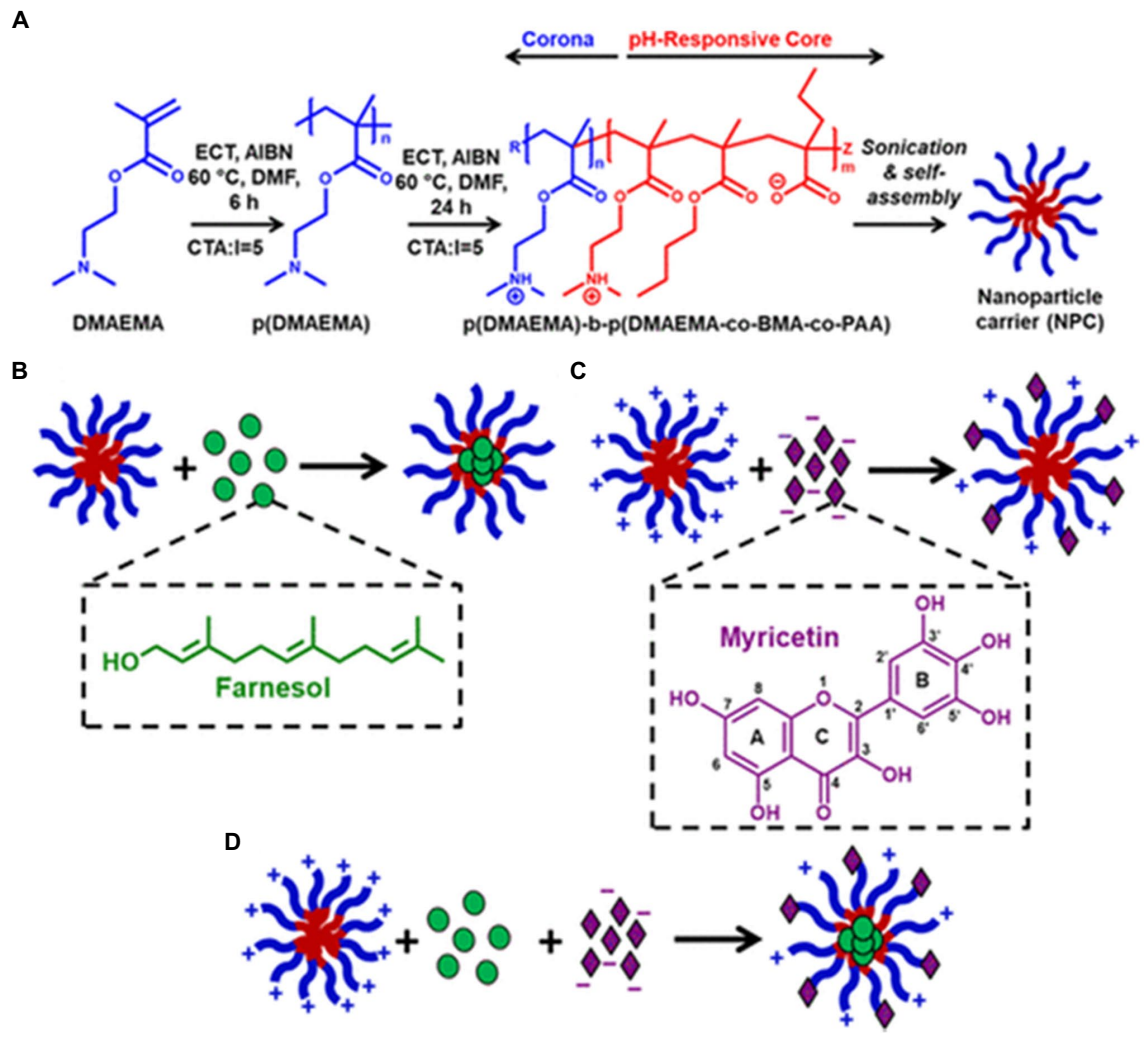
Cationic NPCs hypothesized to coload hydrophobic drugs and flavonoids using different mechanisms. **(A)** Scheme showing the cationic NPC polymer synthesis process, diblock composition, and micelle self-assembly in aqueous conditions; **(B)** Cartoon illustrating the known mechanism of hydrophobic drug (e.g., farnesol) loading within the NPC hydrophobic core; **(C)** Cartoon illustrating the hypothesized mechanism of flavonoid (e.g., myricetin) loading with cationic NPCs via an electrostatic interaction; **(D)** Cartoon illustrating the proposed mechanism of coloading NPCs with farnesol and myricetin. **(A, B)** Reproduced with permission from (27).

Furthermore, nanocarriers with functional groups such as carboxyl and amine groups can increase the solubility of bioactive substances in solvents. These high-density functional groups can be efficiently integrated within the nanocarriers through electrostatic interactions. For example, polymeric nanoparticles with a bilayer load representative flavonoids into deblock polymeric nanoparticle carriers (NPC) DDS with a cationic corona and hydrophobic core. It can improve the problems of poor water solubility and low bioavailability of flavonoids for clinical applications (Figure 7) (83).

### 3.4. Hydrogen bonding

**Figure 8:**
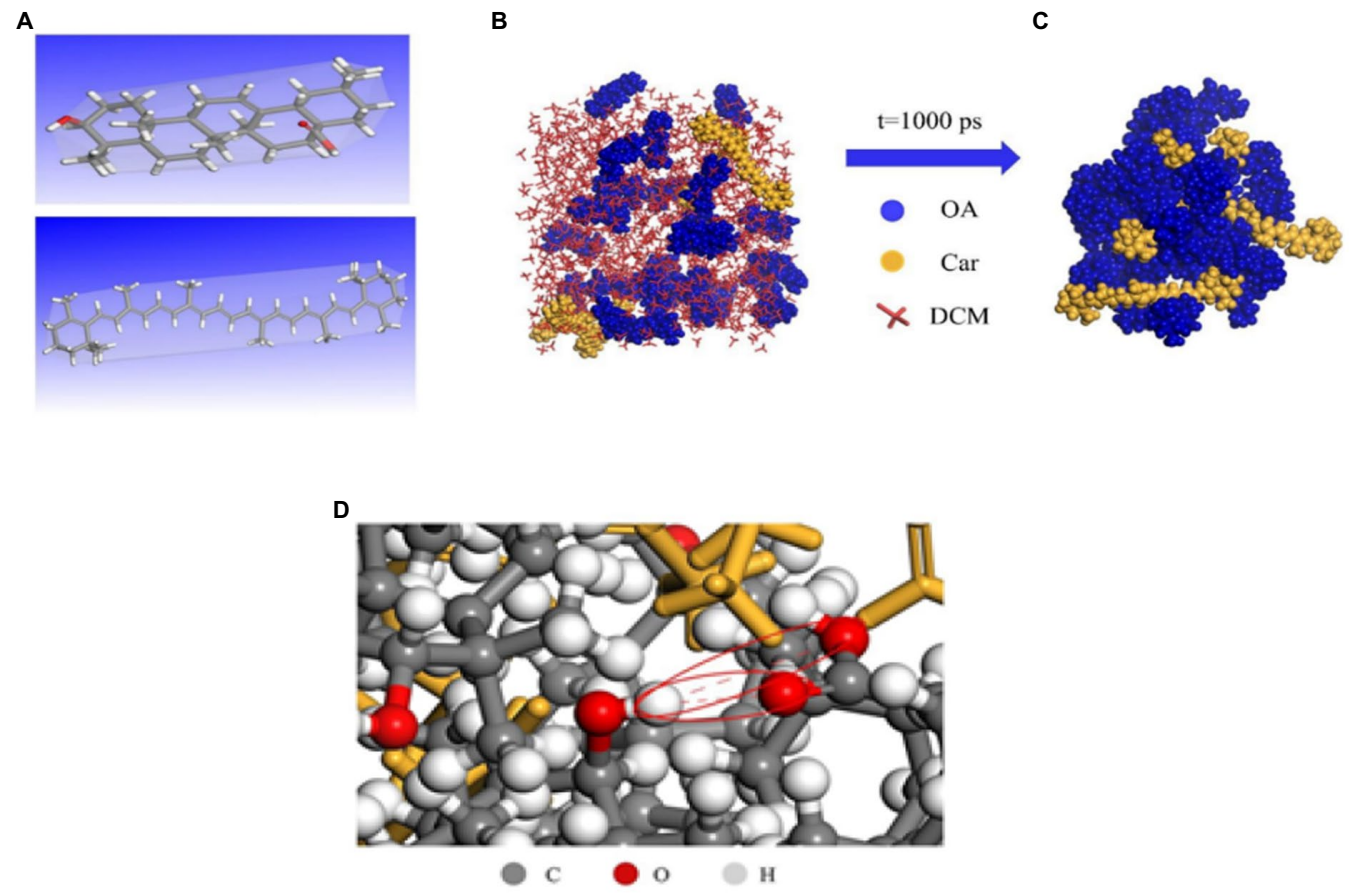
Molecular dynamics (MD) simulation of Car/OA NPs. The optimal geometry of OA (**A**, top), Car (**A**, bottom); OA and Car dispersed in DCM before simulation **(B)**; and Schematic diagram of Car/OA NPs after MD simulation for 1,000 ps **(C)**; Hydrogen bonding in Car/OA NPs **(D)** (84).

Hydrogen bonding is a special type of inter- or intra-molecular interaction force. A hydrogen atom is bonded covalently to an atom X, which has a large electronegativity. If it is close to an atom Y with a large electronegativity and small radius (O F N, etc.), a special intermolecular or intramolecular interaction in the form of X-H.Y is generated between X and Y using hydrogen as a medium, called hydrogen bonding. Hydrogen bonding can combine antibacterial active substance with poor water solubility with macromolecular nanocarriers. β-carotene (Car) has a wide range of physiological effects, but its insolubility in water leads to low bioavailability. Therefore, hydroxyl and carboxyl groups on the surface of carotene can be used to combine with organic functional groups on the surface of oleanolic acid (OA) to form hydrogen bonds (84). As shown in Figure 8, this is a dynamic process of self-assembly of Car and OA hydrogen bonds. Before assembly, both the OA and Car were free to disperse in DCM (Figure 8B). After 1,000 ps simulation, Car was successfully encapsulated (Figure 8C), and two types of hydrogen bonds with a length of 3.562 and 3.347 Å were observed (as shown in red circles in Figure 8D), corresponding to the –C––O···H–O– and –O–Ḥ··O–H, respectively. Therefore, it could be assumed that the self-assembly of OA was driven by hydrogen bonding.

## 4. Application of nano slow-release system

**Figure 9:**
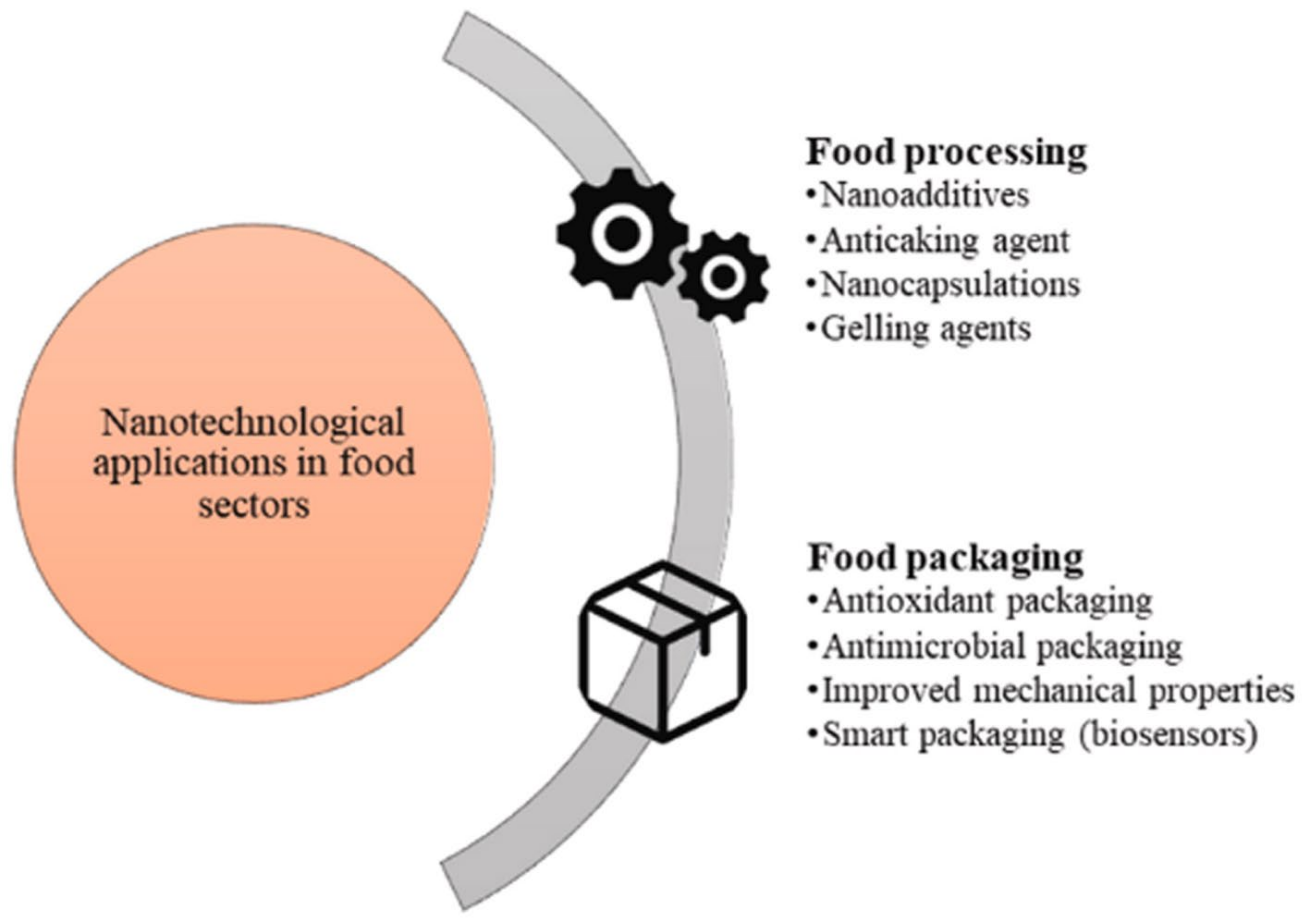
Nanotechnology in Food (85).

Various bioactive compounds are usually poorly stable, water-soluble, and have toxic effects that make it difficult to perform their potential active functions. These drawbacks can be addressed by nano-release systems (e.g., nanoparticles and nano-emulsions) to improve stability and water solubility and mitigate toxicity. Loading and transporting antibacterial active substances through nano-delivery systems can be applied to antibacterial preservation, food packaging, the food industry, etc., greatly expanding the range of applications of the antibacterial active substance (Figure 9) (86).

### 4.1. Application of nano slow-release system in bacterial inhibition and preservation

A natural antibacterial active substances such as curcumin, quercetin, and sulforaphane have superior antibacterial properties and can be applied to food preservation for both antiseptic purposes and without any toxic side effects to the human body. However, the most urgent problem in the field of food preservation is that these antibacterial active substances react with food components, resulting in a decrease in the efficiency of their application in food matrices. Therefore, an antibacterial active substance with antibacterial properties can be encapsulated into nanomaterials to achieve sustained release, prevent undesirable interactions and provide high stability and effective antibacterial activity during food storage (87, 88).

Wang Y et al. (89) studied the antibacterial activity of curcumin nano-capsules using gelatin and porous starch against a variety of foodborne pathogenic and spoilage bacteria such as Gram-negative bacteria (Escherichia coli and Yersinia pestis), Gram-positive bacteria (Staphylococcus aureus, Bacillus subtilis and Bacillus cereus) and fungi (Aspergillus niger, Penicillium nodosum and Saccharomyces cerevisiae). Due to the structural differences in cell membranes, the inhibition efficiency of curcumin was different for different strains of bacteria, and curcumin nanocarriers showed better inhibition for fungi than bacteria and higher inhibition for Gram-positive bacteria than Gram-negative bacteria. This study illustrates that the nano slow-release system can maintain the antibacterial properties of curcumin well, which provides a theoretical basis for the application of curcumin nanocarriers in practical food products.

#### 4.1.1. Application in fruits and vegetables

Fruits and vegetables in the post-harvest storage and transportation process due to temperature, humidity and other external conditions susceptible to oxidation, browning, weight loss and other physiological and biochemical changes, seriously affecting the quality of fruits and vegetables and shelf life (90). In particular, microbial contamination caused by spoilage will not only cause losses but more serious is likely to cause food-borne diseases and health hazards (91).

Therefore, it is possible to combine active substances with nanomaterials to make nanofilms, nano coatings, nano packaging materials and other products. It provides effective help for the further preservation of fruits and vegetables. It can greatly extend the shelf life, and preservation period. Deka et al. (92) prepared sodium chitosan phosphate nanoparticles (CPN) containing curcumin and measured the average particle size of CPN and curcumin-loaded CPN to be 53 nm and 91 nm, respectively, and found in practical applications in fruits and vegetables that curcumin nano preparations released more under acidic pH conditions than under normal pH conditions, while curcumin at trace amounts (0.5 mg/ml) had a higher effect on The curcumin nanoparticles showed inhibitory activity against Gram-positive and Gram-negative bacteria as well as fungi, and the nanoparticles were effective in retaining freshness in foods where acidity increases with time. The antibacterial properties and stability of curcumin were maintained after complexation, and the combination of curcumin and nanomaterials produced an antibacterial agent that is less likely to develop resistance both in application and preservation, which increases the potential of curcumin applications.

#### 4.1.2. Application in dairy products

Antioxidant compounds such as flavonoids are the most important plant active compounds and they have significant health-promoting effects. Direct addition of flavonoids to food, especially to foods such as beverages and dairy products, can lead to undesirable taste and color, while encapsulation with nanomaterials can reduce the loss of bioactive compounds, enhance their dispersion in aqueous solutions, and improve their oral bioavailability. Concerned scholars have added curcumin to milk to reduce the oxidative effect of lipids in milk. Hee prepared a curcumin nanoemulsion (Cur-Nes) that could be added to milk to reduce lipid oxidation, and its free radical scavenging activity did not vary significantly with water content but increased significantly with surfactant concentration (93). *In vitro* lipid, digestibility tests revealed that high surfactant concentration promoted the dissolution of curcumin in the oil phase and increased the slow-release effect. This resulted in increased antioxidant activity, delayed lipid degradation and improved milk quality.

Colloidal formulations containing curcumin are generally available in micelles and hydrogels, and the use of colloidal formulations containing curcumin can be equally effective in increasing its solubility, and stability. The use of colloidal formulations containing curcumin is also effective in increasing its solubility, stability, and slow-release properties, as well as its functionality and antioxidant and antimicrobial activities in food products (94). Esmaili et al. (95) proposed a scheme to load curcumin with protein as a colloidal carrier using proteins from camel milk to make micelles containing curcumin and measured that the solubility of curcumin loading in the colloid increased by at least The solubility of curcumin loading in colloids was measured to be increased by at least 2,500-fold, and it also exhibited more antioxidant properties than free curcumin.

### 4.2. Application in the biomedical field

Some wound dressings currently in use have interesting characteristics such as excellent porosity, good water absorption, moderate water vapor transmission, high drug loading efficiency, and good ability to provide a moist environment, but they are limited in terms of antimicrobial properties (96). They do not protect wounds from microbial invasion, leading to exposure to microbial infection and causing delays in the wound-healing process. In addition, some wound dressings contain synthetic antibiotics that can cause undesirable side effects to the patient. Natural active substances exhibit unique characteristics such as good biocompatibility and reduced toxicity (97). Curcumin is one such natural compound that has demonstrated several biological activities such as anticancer, antibacterial and antioxidant properties. Its good antibacterial and antioxidant activity make it beneficial in the treatment of wounds. Selenia (98) developed a novel wound dressing comprising curcumin-in-liposomes-in-chitosan hydrogel. The prolonged retention time of curcumin is assured by chitosan hydrogel and sustained delivery provided by liposomes-in-hydrogel. The antibacterial ability of curcumin is greatly improved, which provides rapid wound healing.

## 5. Conclusion and prospects

With the improvement of people’s living standards and more and more attention to food health and safety, the demand for food is also increasing to “green” and “natural” change. On the other hand, with the abuse of chemical pesticides, pathogenic microorganisms develop resistance to drugs, endangering the environment and human health. Therefore, it has become a new direction of food safety research to find new natural antibacterial active substances with application value. This paper reviews the construction of a nano-sustained-release system for active antibacterial substances, from the selection of nanomaterials to the innovation of preparation technology. Different nano-carrier materials are selected for loading and encapsulation according to different application ways of active antibacterial substances. Food-grade antibacterial preservation packaging mainly uses green nanocarrier materials such as polysaccharides, porous starch, and protein, which can significantly improve the stability of active antibacterial substances and import safety to ensure food safety. However, fruit and vegetable pathogenic microorganisms can use inorganic and mixed nanocarrier materials. Compared with simple antibacterial substances based on the specificity and specificity of pathogenic microorganisms, there is almost no resistance to nanocarrier materials. Moreover, the nanocarrier materials of the bacteriostatic active substances can gradually destroy the bacterial membrane of bacteria and achieve an excellent bacterial inhibition effect.

Although the research on using nanotechnology to deliver active antibacterial substances has been very extensive, there are still some problems in its practical application based on the instability of active antibacterial substances and the safety of nanocarrier materials. First, the current assessment methods for antibacterial preservation are limited, mainly E. coli and S. aureus, and some indicators of antioxidant tests. Less information is related to fungi, viruses, and other dangerous bacteria. Secondly, the targeted modification of active antibacterial substances based on delivery systems is still challenging to achieve scale-up production and commercial application in practice, mainly because the materials’ low loading, high cost, and potential toxicity restrict the scale-up production. At the same time, the existing preparation process and encapsulation technology methods have particular contingency and irreducibility and lack uniform quality control standards and clinical trial systems.

Therefore, more research is needed to gain a deeper understanding of the interactions between these nanomaterials and active antibacterial substances and to continuously explore and optimize the preparation process of nanomaterials to accelerate the development of more economical, stable, high-utilization, slow-release nanomaterials. It is necessary to strengthen the research on the mechanism of assembly and release of bioactive substances in nanocarriers to clarify the chemical interaction between the substances and carriers. To enhance the research on the assembly and release mechanism of bioactive substances in nanocarriers, clarify the chemical interaction between the substances and the carrier, establish an ideal drug delivery method, and provide a theoretical basis for the prediction and development of the subsequent formulation of drug delivery carriers. This review will inspire researchers to develop and synthesize nano-delivery platforms and technological approaches with various properties and functions to broaden their application range in agriculture, food, and medicine.

## Author contributions

JC and MG: conceptualization and writing – original draft. JW: resources. YL and JL: methodology and formal analysis. XZ and YP: visualization. GC and XH: project administration. DX: funding acquisition. GL: writing – review and editing. All authors contributed to the article and approved the submitted version.

## Funding

This work was supported by National Key Research and Development Program of China (2022YFF0606800), China Agriculture Research System of MOF and MARA (CARS-23-E03), and the Risk Assessment on Vegetable Products(GJFP20210201).

## Conflict of interest

JL was employed by the company Internal Trade Food Science Research Institute Co., Ltd.

The remaining authors declare that the research was conducted in the absence of any commercial or financial relationships that could be construed as a potential conflict of interest.

## Publisher’s note

All claims expressed in this article are solely those of the authors and do not necessarily represent those of their affiliated organizations, or those of the publisher, the editors and the reviewers. Any product that may be evaluated in this article, or claim that may be made by its manufacturer, is not guaranteed or endorsed by the publisher.
